# Metabolomic Profiling Analysis of Physiological Responses to Acute Hypoxia and Reoxygenation in Juvenile Qingtian Paddy Field Carp *Cyprinus Carpio Var Qingtianensis*

**DOI:** 10.3389/fphys.2022.853850

**Published:** 2022-05-20

**Authors:** Yuhan Jiang, Ming Qi, Jinpeng Zhang, Yuanlin Wen, Jiamin Sun, Qigen Liu

**Affiliations:** ^1^ Centre for Research on Environmental Ecology and Fish Nutrition of the Ministry of Agriculture, Shanghai Ocean University, Shanghai, China; ^2^ Key Laboratory of Exploration and Utilization of Aquatic Genetic Resources, Ministry of Education, Shanghai Ocean University, Shanghai, China; ^3^ Shanghai Engineering Research Center of Aquaculture, Shanghai Ocean University, Shanghai, China; ^4^ Zhejiang Fisheries Technical Extension Center, Hangzhou, China; ^5^ Huzhou Academy of Agricultural Sciences, Huzhou, China

**Keywords:** carp *Cyprinus carpio var qingtianensis*, metabolomics, hepatopancreas, hypoxia, reoxygenation

## Abstract

The Qingtian paddy field carp (*Cyprinus carpio var qingtianensis*) is a local carp cultivated in the rice field of Qingtian county, Zhejiang province, China. The paddy field environment is distinct from the pond environment. Due to the inability to artificially increase oxygen, the dissolved oxygen greatly changes during the day. Therefore, investigating the physiological regulation to the changes of acute dissolved oxygen in Qingtian paddy field carp (PF-carp) will dramatically clarify how it adapts to the paddy breeding environment. The high tolerance of Qingtian paddy field carp to hypoxia makes it an ideal organism for studying molecular regulatory mechanisms during hypoxia process and reoxygenation following hypoxia in fish. In this study, we compared the changes of metabolites in the hepatopancreas during hypoxia stress and the following reoxygenation through comparative metabolomics. The results showed 131 differentially expressed metabolites between the hypoxic groups and control groups. Among them, 95 were up-regulated, and 36 were down-regulated. KEGG Pathway enrichment analysis showed that these differential metabolites were mainly involved in regulating lipid, protein, and purine metabolism PF-carps could require energy during hypoxia by enhancing the gluconeogenesis pathway with core glutamic acid and glutamine metabolism. A total of 63 differentially expressed metabolites were screened by a comparison between the reoxygenated groups and the hypoxic groups. Specifically, 15 were up-regulated, and 48 were down-regulated. The KEGG Pathway enrichment analysis supported that PF-carp could continue to gain energy by consuming glutamic acid and the glutamine accumulated during hypoxia and simultaneously weaken the ammonia-transferring effect of amino acids and the toxicity of ammonia. By consuming glycerophospholipids and maintaining the Prostaglandin E content, cell damage was improved, sphingosinol synthesis was reduced, and apoptosis was inhibited. Additionally, it could enhance the salvage synthesis and *de novo* synthesis of purine, reduce purine accumulation, promote the synthesis of nucleotide and energy carriers, and assist in recovering physiological metabolism. Overall, results explained the physiological regulation mechanism of PF-carp adapting to the acute changes of dissolved oxygen at the metabolic level and also provided novel evidence for physiological regulation of other fish in an environment with acute changes in dissolved oxygen levels.

## Introduction

Dissolved oxygen (DO) is one of the most influential environmental factors for aquatic animals. Hypoxia in fish can contribute to adverse effects on growth, reproduction, behavior, and overall survival (Pollock et al., 2007). In life history, dissolved oxygen in water bodies is often influenced by natural factors such as season and region. The level of dissolved oxygen often results in a decline in habitat quality and then affect the migration, growth, and reproduction of some fish populations (Saucier and Baltz, 1993; Comeau et al., 2002; Jin et al., 2019). Some previous studies showed that hypoxia influenced fish growth rate (Bejda et al., 1992; McNatt and Rice, 2004), led to growth retardation, delayed sexual maturation (Diaz Pauli et al., 2017), and restricted the development of fertilized eggs (Heuton et al., 2018).The annual economic loss by DO in the freshwater aquaculture industry is estimated to be billions of US dollars worldwide (Huo et al.,.2018) When fish suffer from hypoxia in aquaculture, increasing the dissolved oxygen in the water is common to alleviate the hypoxic condition.

Fish encountering hypoxic stress is more common in aquaculture, where fish are usually cultured at high densities, limited by environmental conditions and culture patterns, such as rice fields with shallow water. In addition, fish are particularly susceptible to oxidative damage caused by rapid reoxygenation following hypoxic stress (Hirsch et al., 2014). However, due to a lack of systematic research, it is not clear whether the reoxygenation of fish after hypoxia is a simple, reversible physiological process. Some previous studies reported changes in antioxidant enzyme activity, energy metabolism, and other phenotypic physiological parameters (Hughes et al., 1973; Thomas et al., 1988; Muusze et al., 1998). Furthermore, several researchers revealed the regulatory mechanisms of hypoxia adaptation in aquatic animals through transcriptomic, proteomic, metabolomic and other molecular biology techniques (Artigaud et al., 2012; Lai et al., 2016; Chen et al., 2019). However, the entire physiological response and regulatory mechanism of reoxygenation after hypoxia are still unclear.

Metabolomics is the study of the overall profile of endogenous metabolites (low-molecular-weight molecules) present in an organism or biological sample (metabolome) (Casu et al., 2017). It has been successfully applied to disease, nutritional interventions and toxicity studies in fish (Kodama et al., 2014; Ma et al., 2015; Olsvik et al., 2017; Jarak et al., 2018). The rice-fish co-culture system in Qingtian, Zhejiang has been passed down for thousands of years, and it was listed as one of the first batch of Globally Important Agricultural Heritage by FAO in 2005 (Ren et al., 2018). The Qingtian paddy field (*Cyprinus carpio var qingtianensis*) is a specific carp suitable for the system. Currently, the majority of research on the PF-carp focus on skin color, symbiotic relationship between rice and fish, genetic diversity (Xie et al., 2011; Chen et al., 2019; Du et al., 2019). The water environment of rice fields is distinct from traditional aquaculture environment. The main differences are shallow water, large changes in daily dissolved oxygen, and fluctuations in the low oxygen range (DO < 4 mg/L). In addition, there is a risk of drought. Because of these differences above, PF-carp may evolve a strong ability to tolerate hypoxia in the long-term rice-fish co-culture process. However, the mechanism of its adaptation to hypoxia has not been clarified. In the present study, we performed liquid chromatography–mass spectrometry (LC-MS) to detect the changes in metabolites of the hepatopancreas tissue of juvenile PF-carps after acute hypoxia and reoxygenation, and analyzed metabolic pathways in order to further clarify the physiological regulation mechanisms related to acute dissolved oxygen changes from the perspective of phenotypic function execution.

## Materials and Methods

### Experimental Fish, Acute Hypoxia and Reoxygenation Exposure Experiment

Healthy juvenile PF-Carps were transferred from Yugong ecological agricultural technology Co. Ltd. (Qingtian, Zhejiang, China) to the Fisheries Ecology Laboratory at Shanghai Ocean University. Three polyethylene tanks (250-L) were prepared and 9 PF-carps were randomly kept in each tank for 2 weeks prior to the acute hypoxic and reoxygenation experiment. The individual specifications were 56.64 ± 10.74 g in weight, 15.36 ± 1.29 cm in length, and 102 days old. For the first two weeks of the experiment, artificial feed (1% of the fish’s body weight: 30% crude protein content, 3% crude fat content; Techbank, China) was performed once a day at 8:00 and the water was changed once at 18:00 (50% of the volume in the tank). Water temperature was controlled at 25.17 ± 0.41°C and DO at 6.56 ± 0.20 mg/L. Feeding was interrupted the day before the experiment, and all water was replaced; water temperature and dissolved oxygen were checked every 10 min (mins) using a multifunctional dissolved oxygen meter (YSIPro20, United States). To reduce the stress caused by water changes, juvenile PF-carps were allowed to acclimatize for 6 h (hs) prior to the experiment. At the beginning of the experiment, 3 juvenile PF-carps (A total of 9 fish were collected, 6 of which were used for metabolic experiments) were selected from each of the three tanks and euthanized with a concentration of 0.3 mg/L MS-222. Their hepatopancreases were excised and immediately frozen in liquid nitrogen. The samples were transferred to a −80°C freezer. During this procedure, DO was 6.53 ± 0.41 mg/L (hereafter referred to as the control group, CH).

We then rapidly flushed the water in all three tanks with N_2_ to reduce DO levels until the DO was around 0.5 mg/L (about 34 min). The hypoxic treatment was regulated by the flow of N_2_ and O_2_ to maintain a DO concentration of 0.5 mg/L and a water temperature of 25°C. The hypoxic stress experiment lasted for 6 h s, during which DO was averaged at 0.53 ± 0.07 mg/L and temperature was averaged 25.37 ± 0.45°C. The samples collected during the 6 hs were the same as those collected from the CH group (hereafter referred to as the hypoxic stress group, HH) and the results from the metabolic group were defined as HH.

At the end of the hypoxic stress experiment, the injection of N_2_ into the water was stopped and O_2_ was rapidly injected to raise the level of dissolved oxygen in the water of the three tanks until it reached about 7 mg/L (about 29 min). Then, the injection of O_2_ was regulated to maintain a constant high level. The reoxygenation process lasted for 6 h, the DO was averaged 6.64 ± 0.18 mg/L and temperatures was averaged 25.21 ± 0.37°C (hereafter, reoxygenation group, RH). Samples were collected after 6 hs of reoxygenation in the same way as samples from the CH group. The results of metabolomic were defined as RH.

### Sample Collection and Pretreatment

The hepatopancreas samples were removed from −80°C, 6 samples were randomly taken as the metabolomics test and thawed in steps of −80°C→ −20°C → ice water bath. A total of 50 mg of each sample was weighed into EP tubes. After adding 400 µL of the pre-cooled methanol/water (4:1, v/v) mixture to each EP tube, the samples were broken up using a tissue crusher (parameter setting: −20°C, 50 Hz), then thoroughly mixed using a vortex. The samples were left to settle for 30 min at −20°C. The supernatant was then centrifuged (parameter setting: 4°C, 13,000 g, 15 min) and stored in the LC-MS injection vial for subsequent metabolomics analysis. LC-MS analysis performed on Tissue Samples Methanol, Acetonitrile (LCMS grade), and formic acid, (LCMS grade) purchased from Fisher Scientific (Hampton, NH, United States).

### LC-MS Detection.

The LC-MS was performed on a Thermo UHPLC system equipped with a binary solvent delivery manager and a sample manager coupled to a Thermo Q Exactive Mass Spectrometer (Thermo Scientific, San Jose, CA, United States) equipped with an electrospray interface. The parameters of chromatography were as follows: column: Ethylene Bridged Hybrid C18 (100 mm × 2.1 mm, 1.7 μm, Waters, Milford, United States); gradient mobile phase: (A) deionized water containing 0.1% formic acid, (B) acetonitrile/isopropanol (1:1, v/v) mixture containing 0.1% formic acid; flow rate: 0.4 ml/min; sample injection volume: 10 μL; column temperature: 40°C. The mobile phase gradient was: 0–3 min, A: 95–80%; 3–9 min, A: 80–5%; 9–13 min, A: 5–5%; 13–13.1 min, A: 5–95%; 13.1–16 min, A: 95–95%. The MS conditions included the scan ranges (M/Z): 70–1050; sheath gas flow rate (psi): 40; Aus gas flow rate (psi): 10; Aus gas heater temp (°C): 400; normalized collision energy (V): 20–40–60; and IonSpray Voltage Floating (V): positive mode (ESI^+^), +3500; negative mode (ESI^−^), −2800. A QC sample was inserted every 6 analytical samples during the experiment to evaluate the stability of the analytical system and assess the reliability of the results.

### Data Processing and Differential Metabolite Identification

The raw data obtained from the LC-MS analysis of all samples were initially processed using Progenesis QI software (Waters Corporation, Milford, MA, United States). The raw data obtained from the LC-MS analysis of all samples were initially processed using Progenesis QI software (Waters Corporation, Milford, MA, United States). comment HMDB database (http://www.hmdb.ca/), METLIN database (https://metlin.scripps.edu/), KEGG database (https://www.genome.jp/kegg/), and a self-built database were selected for retrieval. Multivariate analyses including principal component analysis (PCA) and orthogonal partial least-squares discrimination analysis (OPLS-DA) were performed by using ROPLS software (v1.6.2). Additionally, the OPLS-DA models were validated using a permutation test with 200 as the permutation number. To select differential metabolites and potential biomarkers, the variable importance in the projection (VIP, VIP >1) values of metabolites in the OPLS-DA model and *P-* values (*p* < 0.05) acquired from the *t*-test analysis were regarded as the screening condition. A KEGG Pathway analysis of the modulated metabolites was performed using Metabo Analyst 4.0 (Chong et al., 2018) of the Metabolomic profiling platform and using Fisher’s exact test. The *p* - values were adjusted by the Benjamini and Hochberg (BH) method. A *P*- value less than 0.05 and Impact Value over 0.1 were selected as the threshold of enrichment significance.

## Results

### Total Ion Map Analysis of Metabolites

**FIGURE 1:**
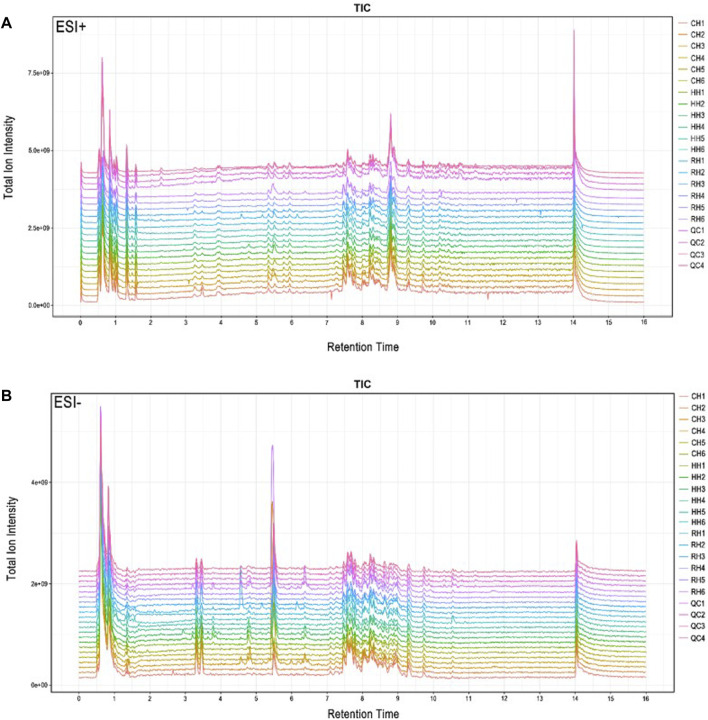
Total ion flow (TIC) of the samples of hepatopancreas of *Cyprinus carpio var. qingtianensis* were obtained in the modes of positive (ESI^+^) and negative (ESI-). Note: **(A)**: ESI + mode; **(B)**: ESI - mode; CH1-CH6: normoxic group with 6 samples; HH1-HH6:6 samples from the experimental group under hypoxic stress for 6 h; RH1-RH6: Reoxygenation recovery with 6 h experiment and 6 samples; QC1-QC4: Quality control with 4 samples.

The typical total ion current chromatograms in positive ion mode (ESI^+^) and negative ion mode (ESI-) of UPLC-Q Exactive are presented in (Figure 1). The overlapped total ion current (TIC) chromatograms of the QC sample demonstrated the strong repeatability of our LC-MS system and an overlap between the response strength and retention time of the chromatographic peak response intensity. This shows that the detection results of UHPLC-Q Exactive analysis platform attain high reliability. A total of 10,168 features were detected at (ESI^+^) ion mode, and 9035 features at (ESI^−^) ion mode.

### Statistical Analysis of Data

**FIGURE 2:**
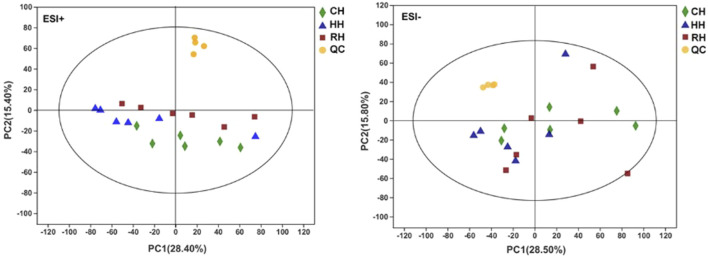
PCA score plot of samples in treatment groups and QC (ESI^+^, ESI^−^).

**FIGURE 3:**
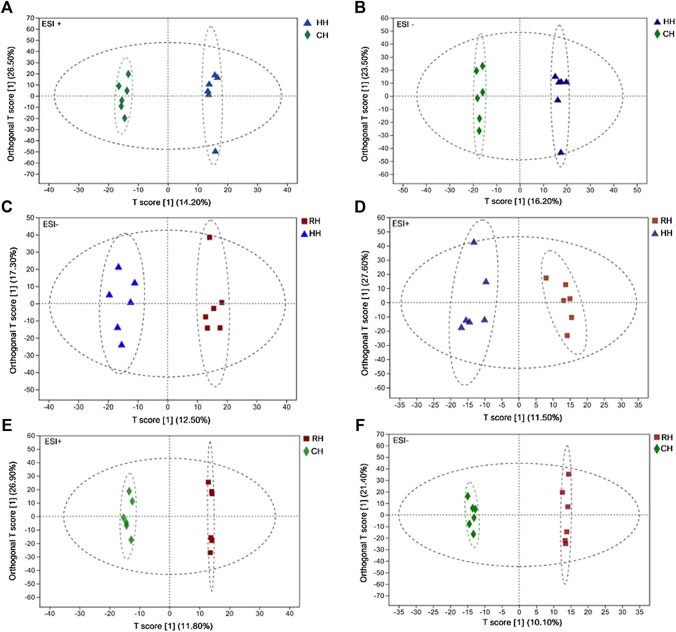
OPLS-DA score scatter plot in HH *vs.* CH, RH *vs.* HH and RH *vs.* CH groups (ESI^+^, ESI^−^) **(A)**: HH *vs*. CH (ESI^+^); **(B)**: HH *vs*. CH (ESI^−^); **(C)**: RH *vs*. HH (ESI^+^); **(D)**: RH *vs*. HH (ESI^−^); **(E)**: RH *vs*. CH (ESI^+^); **(F)**: RH *vs*. CH (ESI^−^).

Quality control (QC) and other experimental samples were analyzed using unsupervised multivariate analysis and supervised analysis (OPLS-DA) after data normalization. Principal component analysis (PCA) was performed on the dataset, and showed that RH groups were under EST^–^, other groups were within the 95% confidence interval (Hotelling’s T-squared ellipse). The *R* ^2^X predictive ability values of the PCA models were 0.570 and 0.541 in positive and negative modes, respectively, suggesting that the data were statistically reliable (Figure 2). Therefore, a PLS-DA model was used to further identify the differences among different groups. The parameter *R* ^2^Y represented the interpretation rate of the model, and *Q* ^2^ represented the prediction rate of the model. Generally, a reliable model requires a parameter higher than 0.4. The results showed the OPLS-DA score plots of the HH *vs.* CH, RH *vs*. HH and RH *vs*. CH groups in positive and negative modes: ESI^+^: *R* ^2^X = 0.994, *R* ^2^Y = 0.964, *R* ^2^Y = 0.997; *Q* ^2^ = 0.616, *Q* ^2^ = 0.522, *Q* ^2^ = 0.696; ESI^–^: *R* ^2^X = 0.995, *R* ^2^Y = 0.974, *R* ^2^Y = 0.997; *Q* ^2^ = 0.746, *Q* ^2^ = 0.593, *Q* ^2^ = 0.569. These reveal that the cumulative *R* ^2^Y and *Q* ^2^ of the OPLS-DA model in ESI^+^ and ESI^–^modes were both above 0.50, suggesting that the models are ideal for prediction and reliability. The sample scatter points between each comparison group are concentrated on both sides of the T score [1] axis, indicating that the obvious differences between the comparison groups and the OPLS- DA model groups (Figure 3). Finally, the screening of differential metabolites between the comparison groups was performed based on VIP values >1 (OPLS-DA model) and *p* < 0.05 (one-way ANOVA).

### Screening of Differential Metabolites

**FIGURE 4:**
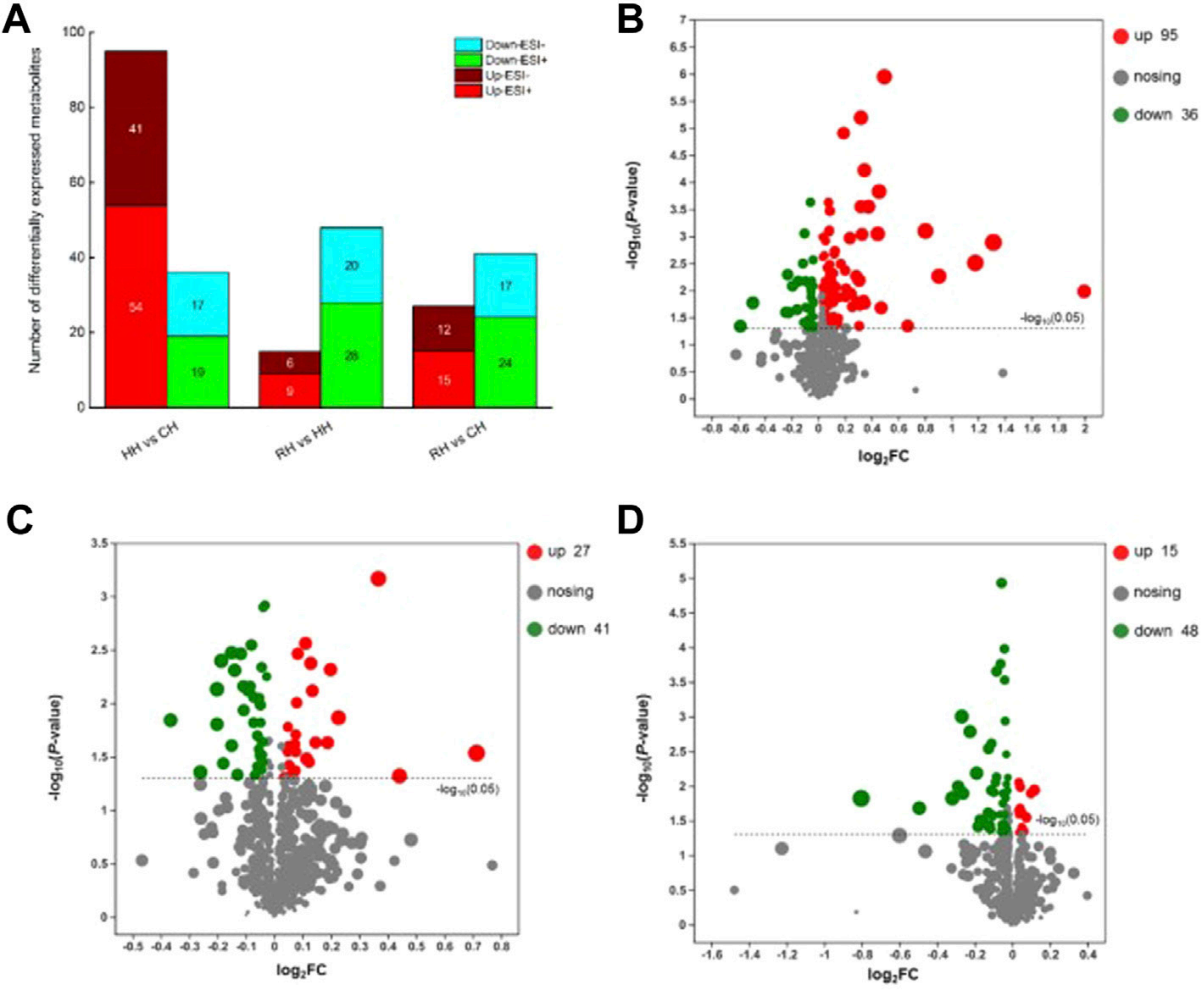
Statistics for metabolites of differential expression among the three comparative groups. Note: **(A)**: Overview of different metabolites between two comparison groups; **(B)**: The volcanic figure of differential expressed metabolites in HH *vs*. CH groups; **(C)**: The volcanic figure of differential expressed metabolites in RH *vs*. HH groups; **(D)**: The volcanic figure of differential expressed metabolites in RH *vs*. CH groups.

To screen the differential metabolites, the VIP in the OPLS-DA model (VIP >1) and *p*-value of Student’s t-test (*p* < 0.05) were used as the criteria. A binding Human Metabolome Database (HMDB, http://www.hmdb.ca/) information search was used to identify differential metabolites. The results showed that 95 up-regulated and 36 down-regulated metabolites were screened and identified in the HH *vs.* CH group, a total of 131 differentially expressed metabolites (DEMs). The detected metabolites mainly included guanosine, D-Ornithine, phosphatidylethanolamine (PE(16:0/22:6(4Z,7Z,10Z,13Z,16Z,19Z))), Sphingolipid metabolism (d18:1/16:0), Sphingosine (Sph), lysophosphatidylcholine (LPC(20:4(5Z,8Z,11Z,14Z))), PE(16:0/20:4(5Z,8Z,11Z,14Z)), Estriol-16-Glucuronide et al. A total of 63 DEMs were screened in RH *vs.* HH, of which 15 metabolites were down-regulated and 48 metabolites were up-regulated. The metabolites mainly included PE (18:1(11Z)/18:1(9Z)), Sph, Valine, Spermidine, Aminoacetone, 3-Hydroxy-N6, N6, N6-trimethyl-L-lysine (TML), Tetrahydroaldosterone-3-glucuronide, hexanoic acid, D-Glutamine, Ethyl beta-D-glucopyranoside, et al. The identification of DEMs in the RH *vs*. CH groups mainly included PE(20:3 (8Z,11Z,14Z)/P-16:0), Phosphocholine, sphingomyelin (SM(d18:1/24:1(15Z))), Glycerophosphocholine (GPC), Gamma-Aminobutyric acid (GABA), L-Methionine, Estriol-16-Glucuronide, phosphatidylcholine (PC(22:4(7Z,10Z,13Z,16Z)/P-16:0)), phosphatidylinositol (PI(16:0/20:3 (5Z,8Z,11Z))), D-Glutamine, N-Acetyl-L-glutamic acid, N6, TML et al. (Figure 4). Of these, most genes were involved in the metabolism in terms of carbohydrate metabolism, lipid metabolism, amino acid metabolism and purine metabolism (Table 1).

**TABLE 1:** Significantly different metabolites.

Metabolic pathway ((KEGG Pathway)	Metabolite	ESI^+/−^	Rt (min)	*m*/*z*	VIP Value	*p*-Value	Up/Down-regulated		
							HH *vs*. CH	RH *vs*. HH	RH *vs*. CH
Carbohydrate metabolism	Oxoglutaric acid	ESI^**-**^	0.8218	191.02	1.07	0.017	↑	—	—
	Glyceraldehyde-3-phosphate	ESI^**-**^	8.17	168.99	1.05	0.042	↑	—	—
Amino acid metabolism	Glutamate	ESI^+^	0.48	148.06	1.81	0.027	↑	↓	—
	Glutamine	ESI^**-**^	2.79	145.06	2.70	0.010	—	↓	↓
	N-Acetyl-L-glutamic acid	ESI^**-**^	0.85	188.06	2.75	0.035	—	—	↓
	Ornithine	ESI^+^	1.67	174.12	1.17	0.042	—	↓	—
Lipid metabolism	PC (14:1(9Z)/20:2(11Z,14Z))	ESI^+^	10.55	756.55	1.29	0.050	↑	—	—
	PC (16:1(9Z)/16:1(9Z))	ESI^+^	1.94	730.54	1.72	0.003	↑	—	—
	PE (14:0/22:4(7Z,10Z,13Z,16Z))	ESI^+^	0.54	781.55	1.33	0.030	↑	—	—
	PE (20:3(5Z,8Z,11Z)/18:1(11Z))	ESI^**-**^	3.20	812.54	2.89	0.000	↑	—	↑
	PS (18:0/22:6(4Z,7Z,10Z,13Z,16Z,19Z))	ESI^+^	11.21	836.54	1.58	0.012	↑	—	—
	LysoPC (15:0)	ESI^+^	7.67	482.32	1.27	0.017	↓	—	—
	LysoPC (20:2(11Z,14Z))	ESI^+^	7.16	548.37	1.73	0.031	↑	—	—
	LysoPC (22:4(7Z,10Z,13Z,16Z))	ESI^+^	10.80	572.37	1.75	0.005	↓	↑	—
	PGE-Prostaglandin	ESI^+^	7.31	367.28	2.11	0.032	↑	—	—
	SM (d18:1/16:0)	ESI^+^	1.47	703.57	1.54	0.039	↑	—	—
	Sphingosine	ESI^+^	10.89	300.29	1.42	0.013	↓	↑	—
Purine metabolism	Guanosine	ESI^+^	3.59	284.10	2.80	0.001	↑	↓	—
	Guanine	ESI^+^	0.84	152.06	1.06	0.016	↑	↓	—
	Xanthine	ESI^+^	7.58	153.04	1.02	0.031	↓	—	—
	Glutamate	ESI^+^	0.48	148.06	1.81	0.027	↑	↓	—

Note: a: PC—Phosphatidylcholine, PE—Phosphatidylethanolamine, LysoPC—Lyso phosphatidylcholine, SM—Phingomyelin; b: Variable importance in the Projection (VIP) is obtained from the OPLS-DA, model. These discriminating metabolites were obtained using a statistically significant threshold of variable influence on projection (VIP >1.0). c: *p* value obtained from analysis of variance (ANOVA). Setting the screening threshold to *p*-value < 0.05. *p*-value = 0.000 means *p*-value is less than 0.001; d: “↑” indicates a significant increase, “↓” indicates a significant reduction, “—” indicates no significant difference.

### Differential Metabolites Pathway KEGG

**FIGURE 5:**
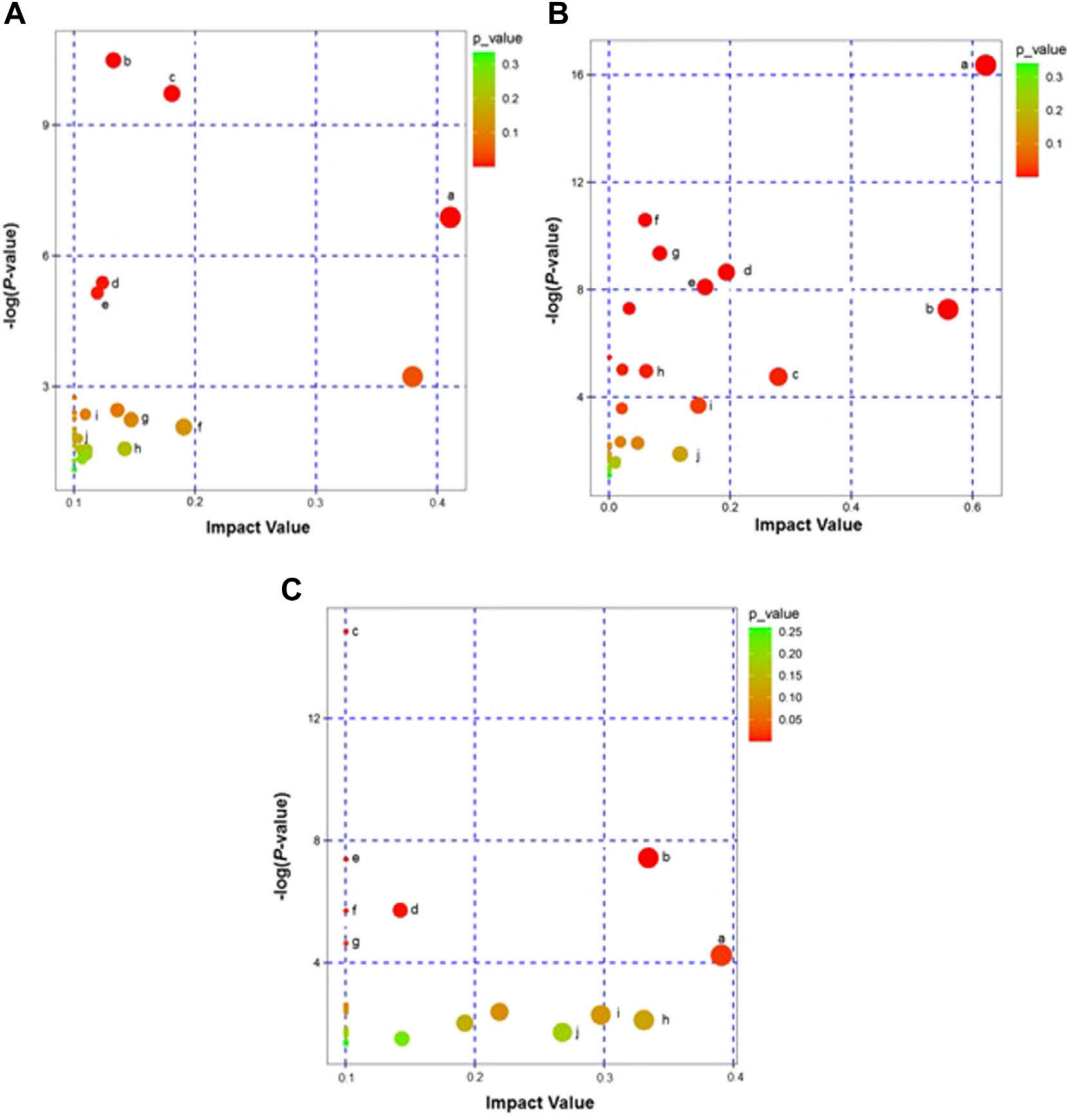
**(A)**: Enrichment of metabolic pathways of DEMs in HH *vs*. CH groups (KEGG Topology Analysis). Note: a: D-Glutamine and D-glutamate metabolism; b: Glycerophospholipid metabolism; c: Steroid hormone biosynthesis; d: Sphingolipid metabolism; e: Purine metabolism; f: Alanine, aspartate and glutamate metabolism; g: Pentose and glucuronate interconversions; h: Glutathione metabolism; i: Linoleic acid metabolism; j: Arachidonic acid metabolism. **(B)**: Enrichment of metabolic pathways of DEMs in RH *vs*. HH groups (KEGG Topology Analysis). Note: a: D-Glutamine and D-glutamate metabolism; b: D-Arginine and D-ornithine metabolism; c: Alanine, aspartate and glutamate metabolism; d: Arginine metabolism; e: Glutathione metabolism; f: Aminoacyl-tRNA biosynthesis; g: beta-Alanine metabolism; h: Glyoxylate and dicarboxylate metabolism; i: Taurine and hypotaurine metabolism; j: Pentose and glucuronate interconversions. **(C)**: Enrichment of metabolic pathways of DEMs in RH *vs*. CH groups (KEGG Topology Analysis). Note: a: Lysine degradation; b: Glycerophospholipid metabolism; c: Alanine, aspartate and glutamate metabolism; d: Aminoacyl-tRNA biosynthesis; e: D-D-Glutamine and D-glutamate metabolism; f: Valine, leucine and isoleucine biosynthesis; g: Pantothenate and CoA biosynthesis; h: Glutathione metabolism; i: Glycine, serine and threonine metabolism; j: Pyrimidine metabolism.

KEGG metabolic pathway analysis showed that the 131 differential metabolites in the HH vs. CH group involved 26 metabolic pathways that mainly relate to the D-Glutamine and D-glutamate metabolism; alanine, aspartate and Alanine; aspartate and glutamate metabolism; sphingolipid metabolism; glutathione metabolism and pentose and glucuronate interconversion; and glucuronate interconversions (schematic representation of important metabolic pathways (Figure 5A). The 63 differential metabolites in the RH *vs*. HH group (of which ESI^+^:11, ESI^−^:4) were involved in 29 metabolic pathways, mainly focusing on the D-glutamine and D-glutamate metabolism; alanine, aspartate and glutamate metabolism; Alanine, aspartate and glutamate metabolism; taurine and hypotaurine metabolism; glyoxylate and dicarboxylate metabolism; D-arginine metabolism. Metabolism; and D-arginine and D-ornithine metabolism (schematic representation of important metabolic pathways (Figure 5B).

In the RH *vs*. CH group, the 68 differential metabolites (ESI^+^:16 (ESI^−^:6)) involved 24 metabolic pathways, mainly involving Lysine degradation, Glycerophospholipid metabolism, Alanine, aspartate and glutamate metabolism, Aminoacyl-tRNA biosynthesis, D-Glutamine and D-glutamate metabolism, and glutamate metabolism (for a schematic representation of important metabolic pathways, see Figure 5C).

## Discussion

Fish are aerobic organisms and dissolved oxygen is one of the most important environmental factors limiting their survival (Abdel-Tawwab et al., 2019). Dissolved oxygen in the water directly affects energy metabolism, blood parameters, and physiological defense against oxidative stress in fishes, et al. (Wu, 2002). In fish, liver (PF-carp for hepatopancreas) are critical to metabolism (Guilherme et al., 2012; Qi M et al., 2020). Changes in dissolved oxygen affect the physiological function of liver and disturb energy metabolism, antioxidant stress and the synthesis of other substances (Almeida-Val et al., 2000; Wang and Richards, 2011; Jung et al., 2014). Furthermore, how liver (hepatopancreas) adapt to the reoxygenation following hypoxia and related molecular regulatory mechanisms remain unclear. Therefore, it is significant to investigate the changes in the metabolome of liver during hypoxia and reoxygenation.

**FIGURE 6:**
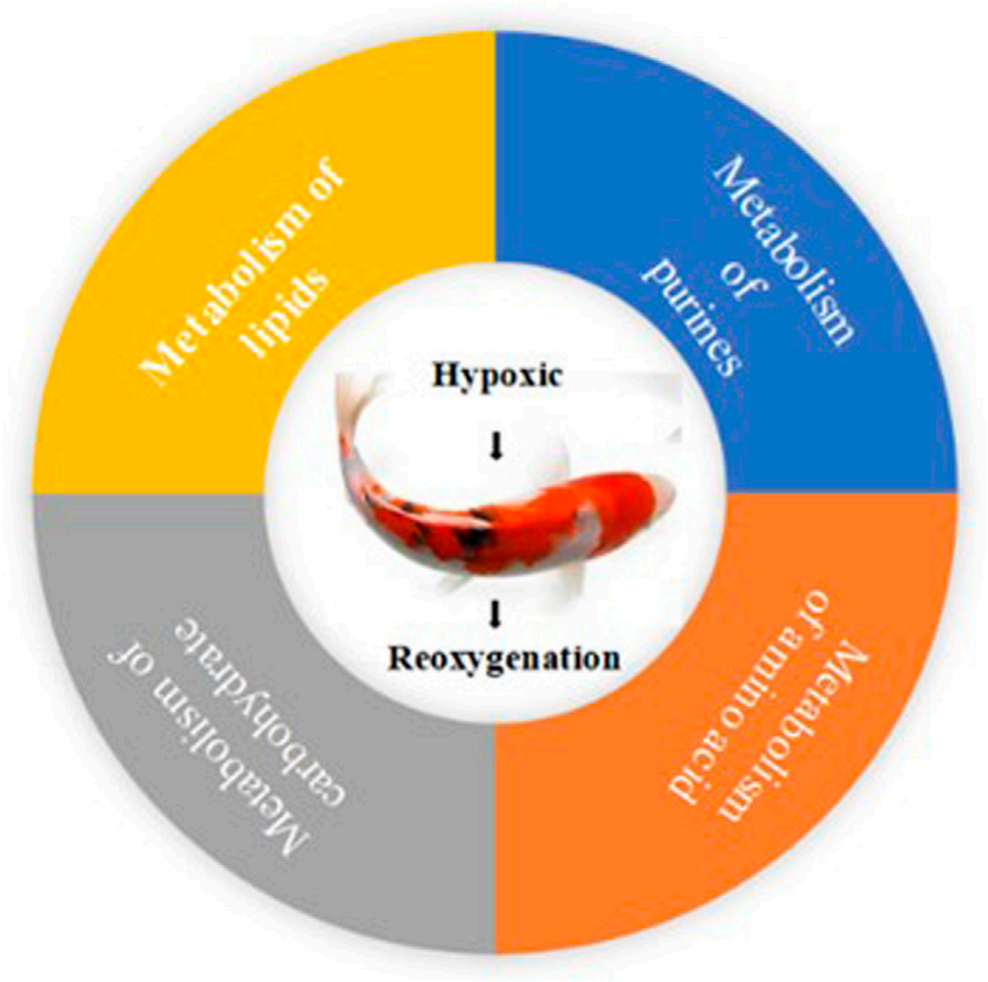
The main metabolites and their related KEGG pathways.

In the present study, LC-MS were performed to screen metabolites in the hepatopancreas of PF-carp during hypoxia and reoxygenation, and KEGG pathway enrichment analyses were then performed. Most genes were involved in metabolisms such as carbohydrate metabolism, lipid metabolism, amino acid metabolism and purines metabolism. The same metabolic pathways were all activated among three groups, including GPs metabolism, glutamine and glutamate metabolism and purine metabolism (Figure 6). To study whether the same metabolic pathway takes part in the same physiological metabolic function in different groups, we analyzed the pathway regulation direction through metabolic pathways and the different metabolites of important nodes. Herein, some of the major metabolites and their related KEGG pathways were discussed below.

### Carbohydrate Metabolism

KEGG enrichment analysis revealed that many DEMs were associated with pathways involving carbohydrate biosynthesis. Fish using the carbohydrate metabolism as the energy-supplying metabolism had the strongest metabolism (Jobling, 1994), and total hepatopancreas glycogen was about 10% of muscle glycogen (Moyes and West, 1995). When the body needs an ample energy supply, liver glycogen will soon be exhausted (Wiseman and Vijayan, 2011), and the sugar metabolism products downstream will also decrease. In our study, important node metabolite of the energy supply pathway from carbohydrate metabolism merely was oxoglutaric acid, and content significantly increased after 6 hs of hypoxia stress. In contrast, other related metabolites showed no significant changes. On the one hand, this may be related to reactive oxygen species (ROS) generated in the low-oxygen stress environment (Choi et al., 2015), which influences the entire metabolism of glycogen and glucose. On the other hand, the capacity of hepatic glycogen stores is limited. Six hours of hypoxia stress caused massive consumption of liver glycogen, but hepatic glycogen is important for the physiological homeostasis of fish (Speers-Roesch et al., 2013). When the liver glycogen is consumed to a certain extent, an amount of glycogen is retained for homeostatic regulation, which eventually leads to an insufficient glucose energy supply and affects the carbohydrate metabolism. After 6 h low-oxygen stress, the content of Cortisone in the hepatopancreas of PF-carp is significantly increased, as cortisol can increase gluconeogenesis (Tintos et al., 2008) when a sugar metabolic is substrate-insufficient, which can convert non-sugar substances into sugar metabolic substrate. One of the important gluconeogenesis pathways is amino acid transamination to form ketoglutarate, which also explains why the ketoglutarate contents will be significantly increased when the glycome supply is not enough (Rosen and Milholland, 1963). After 6 h of reoxygenation, the cortisol content was significantly reduced, but the normoxia group showed a significant increase. It can be inferred that during the reoxygenation recovery stage, the gluconeogenesis in the hepatopancreas of PF-carp was weakened but still in an activated state, so the content of ketoglutarate was not significantly reduced. In a further detection of carbohydrates, it was found that the glyceraldehyde 3-phosphate content was only significantly up-regulated under hypoxic stress in hepatopancrea; however, the other sugar detected did not markedly change during glycolysis and no activation glycolysis pathways were found. This result indicates that the glycolysis pathways is repressed as a whole, and it was detected to be significantly elevated, which may be related to the acetoacetyl-CoA produced by the metabolism of lipids (Kalyananda et al., 1987). In conclusion, PF-carp will weaken the metabolism of saccharides during acute hypoxia stress and reoxygenation recovery through the gluconeogenesis pathway to obtain more energy metabolism substrates, and this process, besides providing energy during early stages of hypoxic stress, may be mainly used to maintain physiological homeostasis later in the experiment.

### Amino Acid Metabolism

During the conversion of proteins, carbohydrates and lipids, the most important link is transamination. There is a large amount of alanine aminotransferase (ALT) in the liver which transfers the amino group from alanine to ketoglutarate for the synthesis of pyruvate and glutamate (Siest et al., 1975). Meanwhile, pyruvate from other tissues of the body (such as muscle) is used to synthesize alanine under the action of transamination, and it enters the liver as a form of pyruvate to be used in the metabolism of pyruvate and nitrogen (Rosenberg, 1946; Felig, 1973). Furthermore, there is also glutamate dehydrogenase (GIDH) in the hepatopancreas which can catalyse the interconversions of glutamate and ketoglutarate according to the energy demand for metabolism and provide the reducing electron carrier NADH. When the energy metabolism substrate is insufficient, GIDH can catalyze the oxidative deamination of glutamate to form ketoglutarate, and then enter the TCA cycle for energy metabolism, finally produce NADH (approximately 2.5 ATP for energy supply) for the energy metabolism substrate. When sufficient, GIDH catalyzes the incorporation of free NH_4_^+^ into the carbon skeleton of ketoglutarate to form glutamic acid (Yang et al., 2009). Glutaminase is the main regulator enzyme of the hepatic glutamine catabolism, which can catalyze the conversion of glutamine to glutamate and ammonia (Simon et al., 2020). Glutamine is catalyzed by glutamine synthase (GS) to form glutamine, which can effectively remove ammonia toxicity (Wee et al., 2007; Hakvoort et al., 2017). In this experiment, the content of three metabolites, glutamate, glutamine, and ketoglutarate, significantly increased after 6 hs of hypoxia stress, which verified the gluconeogenesis of glutamate and glutamine. At the same time, metabolic pathways of arginine and proline are also activated under hypoxia stress, and arginine and proline converted to form glutamic acid for supplementation. After 6 hs of reoxygenation recovery, the content of glutamate and glutamine decreased to the level of the normoxia group, while the content of ketoglutarate did not significantly decrease and was significantly higher than the level of the normoxia group, indicating that glutamate and glutamine continuous re-supply to the carbohydrate metabolism or d other pathways for transamination. When glutamate and glutamine are weakened, glutamate receptors are broadly divided into metabotropic and ionotropic types (Ruiz et al., 2021). Ionotropic glutamate receptors could open ion channels on cell membranes, causing cell ion disturbances, especially Ca^2+^ influx (Dohare et al., 2015). By inducing late-onset injury, excessive concentrations of glutamate require catabolism consumption and conversion through other pathways. Additionally, due to an excess of transamination, NH_4_ is produced in the liver, and NH_4_ has been shown to be toxic to fish (Benli et al., 2008). In this study, it was found that Ornithine and N-Acetyl-L-glutamic acid levels were significantly increased between the hypoxia stress 6 hs group and the normoxia group. N-acetyl-L-glutamic acid is used as a cofactor of carbamoyl phosphate synthase I (Chapel-Crespo et al., 2016) which promotes carbamyl phosphate synthetase I to catalyze glutamate, NH_4_, CO^2^ and water to generate carbamyl phosphate, and enter ornithine and arginine metabolism pathway. The node of cycle is arginine which generates ornithine and urea under the action of arginase (Huggins et al., 1969). When reoxygenation is restored, the contents of Ornithine and N-Acetyl-L-glutamic acid in the hepatopancreas and pancreas of PF-carp are significantly reduced, which also confirms the reoxygenation weakening of transamination in the process.

### Lipid Metabolism

The cell membrane is critical to most enzymes localization and intracellular material metabolism. GP, a membrane component, is very important for metabolism and signal transduction (Parsons et al., 2013). Common GPs include phosphatidylcholine (PC, lecithin), PE, phosphatidylserine (PS). It has been shown that fish have a certain ability to synthesize GP by themselves (Hvattum et al., 2000).

Different metabolite enrichment metabolic pathways showed that the two types of PC (16:1(9Z)/16:1(9Z) and PC (14:1(9Z)/20:2(11Z,14Z)), two types of PE (14:0/22:4(7Z,10Z,13Z,16Z), PE (20:3(5Z,18Z),11Z)/18:1(11Z))) and PS(18:0/22:6 (4Z,7Z,10Z,13Z, 16Z,19Z) content was significantly increased after 6 hs hypoxia stress, which indicates that after being stimulated by hypoxia, the hepatopancreas of PF-carp synthesizes more GP to repair the oxidative damage of the cell membrane. At the same time, the ammonia toxicity produced by transamination also stimulates the synthesis of GP to a certain extent. After 6 hs of reoxygenation, the contents of the above five GPs were significantly reduced, but were significantly higher than the level of the normoxic group, indicating that the synthesis of GP in PF-carp was inhibited and the degradation was accelerated during the reoxygenation The results indicated that the degradation of cell membrane caused by oxidative stress could be improved to some degree. There is a dynamic balance between synthesis and decomposition in the metabolism of GP. One of the most important catabolic pathways is the production of arachidonic acid (ARA) under the action of phospholipase A2 (PLA2), linolenic acid (LA) and lysophosphatidylcholines (LysoPCs). The metabolism of GP shows a dynamic balance between synthesis and decomposition, an important catabolic pathway is the production of ARA (Balsinde et al., 2002), LA(Ballou and Cheung, 1985) and LysoPCs under the action of PLA2. In this study, four LysoPCs were detected (22:4(7Z,10Z,13Z,16Z); 20:2(11Z,14Z); (20:3 (5Z,8Z,11Z); 15:0)). A trend upon hypoxia-induced downregulation and reoxygenation-induced upregulation was observed, which indicates that the metabolism of GPs in the process of hypoxia stress mainly promoted synthesis and inhibited decomposition. To a certain extent, this increases the risk of fatty hepatopancreas forming in the hepatopancreas and pancreas of PF-carp (Zhao et al., 2018). During hypoxia stress, PF-carp activated the ARA and LA metabolic pathways. Prostaglandin E (PGE) content increased after hypoxia stress, indicating PF-carp could synthesize more PGE to improve the immunity and damage-repair ability of hepatopancreas and pancreas tissues during hypoxia. Although the metabolic pathways of ARA and LA were not activated after reoxygenation recovery, the PGE content did not significantly decrease, which implies that the hepatopancreas of PF-carp still have a certain degree of damage after reoxygenation recovery, and PGE continues to exert immunity and damage repair functions. The study also found there are significant changes in sphingolipid (SM) during acute hypoxia stress and reoxygenation. SM can be hydrolyzed by Sphingomyelinase (SMase) to generate phosphocholines and Ceramide. Ceramide is catalyzed by Ceramidase (CDase) to generate Sph and free fatty acids (Hannun and Obeid, 2008). Ceramide can act as a second messenger to inhibit PkC activity by regulating the TNF pathway (tumour necrosis factor pathway) (Spiegel and Merrill, 1996). It interferes with the normal periodic activities of cells (such as differentiation, apoptosis and proliferation) (Ruvolo, 2003). Alcohol is the basic skeleton of intracellular sphingolipids, which can be phosphorylated to produce sphingosine 1 phosphate (S1P) under the action of sphingosine kinase. Studies have shown that the process by which Sph phosphorylation produces S1P can effectively inhibit cell apoptosis, so Sph is often used as a negative regulatory marker of the apoptosis signaling pathway (Cuvillier, 2002). The results of this study showed that after 6 hs of hypoxic stress, the content of Sph was significantly reduced but the content of SM was significantly increased. This indicates that hypoxia stress promoted hepatopancreas cells to inhibit SMase activity, reduced the production of ceramide, and promoted cell apoptosis by enhanced PkC activity; at the same time, the significant decrease in Sph content also confirms accelerated apoptosis of hepatopancreas cells.

### Purine Metabolism

Besides being essential in DNA and RNA synthesis, purines are important components of several biomolecules associated with the energetic metabolism such as ATP, GTP, cAMP and NADH (Peixoto et al., 2019). In the process of DNA and RNA degradation, guanosine and adenosine are released through nucleotidase or phosphatase hydrolysis; during the degradation of GMP and AMP, AMP is not easily catalyzed by nucleotidase. Purine generates GMP and hypoxanthine nucleic acid (IMP) under the action of adenylate deaminase. GMP is then transformed into Guanosine by nucleotidase. A high amount of energy is consumed after hypoxia stress; ATP and GTP are decomposed into AMP and GMP. The ROS brought on by hypoxia stress can also damage nucleic acids and other macromolecular substances and cause their degradation. This explains why the guanosine content in the hepatopancreas and pancreas of PF-carp significantly increased after hypoxia stress. At the same time, studies showed that guanosine could participate in the oxidation reaction and the regulation of glutamatergic parameters during hypoxia (Hansel et al., 2015). Combined with the increased toxicological effects of glutamate after hypoxia stress, the increase in guanosine content under hypoxia is also a regulating mechanism to relieve damage caused by excessive glutamate content. In this study, hypoxic stress can cause hypoxia in the hepatopancreas and pancreas tissues. Due to the inability to supply sufficient molecular oxygen, only two metabolites of guanine and xanthine were detected to be significantly increased in this study. Purine metabolism decreased due to the insufficient supply of molecular oxygen, so no significant increase was detected in uric acid. During the reoxygenation recovery phase, the content of guanosine and guanine was found to be significantly down-regulated, but no significant changes were detected in uric acid. At the same time, glutamine activated the purine metabolism pathway. This is because a high number of purines are decomposed in the hypoxia process, and there is an urgent need for synthetic annulus to form new RNA, DNA and for the synthesis of high-energy phosphate bond carriers. There are two purine nucleotide synthesis pathways in the body: one is the *de novo* synthesis pathway with glutamine, aspartic acid, glycine, phosphoribose one carbon unit and CO_2_ as a substrate; the other is nucleoside and free base. The base is a remedial synthesis pathway catalyzed by adenine phosphoribosyltransferase (APRT) and hypoxanthine-guanine phosphoribosyltransferase (HPRT) (Schramm and Bagdassarian, 1999).

## Conclusion

In this study, we used LC-MS technology to compare the metabolic levels of hepatopancreas during hypoxia stress and the following reoxygenation through a comparison of metabolites. It was found that during the process of hypoxia stress, PF-carp enhanced the gluconeogenesis after amino acid transamination by taking the core metabolism of glutamate and glutamine, replenishing the intermediate products of the energy metabolism, and enhancing glutamine and removing ammonia toxicity through enhanced glutamine synthesis and urea cycling. This improves immunity and antioxidant capacity by enhancing the metabolism of phospholipids and sphingolipids and clears damaged cells by accelerating apoptosis; the purine metabolism is also enhanced to remove damaged nucleotides, and produce guanosine to antagonize the toxicological effects of glutamate, assisting the completion of hypoxic adaptation.

The continuous energy supply produced by the consumption of glutamate and glutamine accumulated during hypoxia stress could inhibit the transamination of amino acids and weak the poison of ammonia. It could also maintain the content of PGE to repair cell damage and reduce the synthesis of Sph, and inhibit cell apoptosis during reoxygenation recovery. Through supplement and *de novo* synthesis of purines, the synthesis of nucleotides and high-energy phosphate bond carriers were promoted, and the damage was inhibited. Notably, the carbohydrate metabolism does not significantly change during this process, which may be related to the rapid consumption of hepatopancreas glycogen. After being consumed to a certain extent, it will no longer be consumed, but instead is used to maintain the body’s homeostasis. Our paper explains the adaptation and regulation mechanism of the physiological metabolism of PF-carp at the metabolic level during acute dissolved oxygen changes. However, follow-up analysis needs to be combined with other omics to screen important and special metabolic pathways for providing an accurate explanation of the physiological regulation mechanism upon PF-carp during acute dissolved oxygen changes Cheek, 2011, Diaz, 2001, Small et al., 2014, Chen et al., 2019.

## Data Availability

The original contributions presented in the study are included in the article/Supplementary Material, further inquiries can be directed to the corresponding authors.
